# The Impact of Green Space on Violent Crime in Urban Environments: An Evidence Synthesis

**DOI:** 10.3390/ijerph16245119

**Published:** 2019-12-14

**Authors:** Mardelle Shepley, Naomi Sachs, Hessam Sadatsafavi, Christine Fournier, Kati Peditto

**Affiliations:** 1 Department of Design & Environmental Analysis, Cornell University, Ithaca, NY 14850, USA; nsachs@healinglandscapes.org (N.S.); ksp66@cornell.edu (K.P.); 2 Department of Plant Science and Landscape Architecture, University of Maryland, College Park, MD 20742, USA; 3 Department of Emergency Medicine, University of Virginia School of Medicine, Charlottesville, VA 22908, USA; hs8pb@virginia.edu; 4 Mann Library, Cornell University, Ithaca, NY 14850, USA; ctf43@cornell.edu

**Keywords:** violent crime, urban parks, greenspace, green space, scoping review, systematic review, literature review

## Abstract

Can the presence of green space in urban environments reduce the frequency of violent crime? To ascertain the evidence on this topic, we conducted an in-depth literature review using the PRISMA checklist. The search parameters included US articles written in English and published since 2000. More than 30,000 potential paper titles were identified and ultimately, 45 papers were selected for inclusion. Green spaces typically comprised tree cover, parks and ground cover. Criminal behaviors typically included murder, assault, and theft. The majority of the research reviewed involved quantitative methods (e.g., comparison of green space area to crime data). We extracted multiple mechanisms from the literature that may account for the impact of green space on crime including social interaction and recreation, community perception, biophilic stress reduction, climate modulation, and spaces expressing territorial definition. Recommendations are made for future research, such as meta-analysis of existing data and the development of grounded theory through qualitative data-gathering methods. By providing evidence that access to nature has a mitigating impact on violence in urban settings, city governments and communities are empowered to support these interventions.

## 1. Introduction

In this literature review, we investigate whether the presence of nature in urban environments reduce the frequency of violent crime. Research suggests that, in many circumstances, green space in the form of trees, parks, and other natural areas, may have a mitigating impact. By providing evidence that the presence of nature contributes to the reduction of violence in urban settings, city governments and communities will be empowered to support these interventions.

The positive impact of nature and green space on human health and well-being has been documented by over 100 studies [1,2,3], including several literature reviews and meta-analyses which have examined the benefits of the nature connection [2,4,5,6,7,8,9,10]. Several researchers have begun to explore the relationship between nature and urban crime, focusing on outcomes such as reduced aggression and improved community cohesion [11,12,13,14]. Multiple new papers and dissertations have been published in the last three years [15,16], and an expansive update is essential to setting future research agenda.

The following paper synthesizes the evidence of the impact of green space on violence by utilizing methods from systematic and scoping reviews. We are both addressing a specific question and describing the broader literature. A systematic review incorporates “appropriate” study designs that are pre-identified and include a paper quality assessment, which the research in this paper also undertakes [17]. A scoping review shares similar methods and aims, although the scoping review is concerned with presenting the characteristics of existing literature on a topic, whereas a systematic review aims to summarize the “best available research” on a topic [18].

Although violent crime in the United States has fallen since 1997 [19], Grinshteyn and Hemenway found that in 2010, the US gun homicide rate was 25 times higher than the rate in other high-income countries despite a similar rate of nonlethal crimes [20]. The study also reported that Americans are ten times more likely to die by firearms compared with residents of other countries. While many of these crimes are homicides, approximately 60% are suicides [21]. Additionally, there has been an increase in mass shootings. In 1994–2004, the average annual rate of mass shootings was 1.12 shooting per 100 million, while in 2005–2013, the average annual rate was 1.41 shooting per 100 million [22].

### 1.1. Definitions

According to Pati and Lorusso [23], one of the major challenges in conducting a systematic literature review is identifying the search terms. In this paper, the two primary categories were “green space” and “violent crime.”

*Greenspace*. Green space is defined as “synonymous with nature” and “explicitly urban vegetation” [24]. In reviewing a range of journals, the authors identified many related terms including garden, ecological garden, urban forest, urban parks, urban habitat, greenery, greenbelt, green area, green environments, green network, green infrastructure, natural environment, parkland, walkable area, blue space, green patches, riparian greenspace, sky garden trees, urban farm, urban ecosystem, water bodies, woodland, and vegetated areas. This study uncovered six definitions of green space in the literature:

- Vegetation, ranging from sparsely landscaped streets to tree-lined walkways to playfields and forest parks [25].
- Combined areas of open land, cropland, urban open land, pasture, forest, and woody perennial [26].
- Land use that has notable contributions to urban environments in terms of ecology, aesthetics, or public health, but which basically serves human needs and uses [27].
- Areas with substantial green elements [28].
- Recreational or undeveloped land [29].
- Predominantly covered with vegetation [30].

Informed by this analysis, we defined green space using the broad description provided by the US Environmental Protection Agency (EPA). According to the EPA, green space is “land that is partly or completely covered with grass, trees, shrubs, or other vegetation… Green space includes parks, community gardens, and cemeteries” [31].

*Violent crime*. The U.S. Department of Justice Federal Bureau of Investigation, in their Uniform Crime Reporting (UCR) program, defines violent crime as “composed of four offenses: murder and nonnegligent manslaughter, forcible rape, robbery, and aggravated assault. Violent crimes are defined in the UCR Program as “those offenses which involve force or threat of force” [32].

### 1.2. Goals

We have three goals associated with this research. First, to assess through a literature review where we stand with regard to studies that address the question: Can the presence of nature in urban environments reduce violent crime? Second, to generate an agenda for future research based on the gaps that are identified as part of this assessment. Lastly, to explore the mechanisms that might account for the interaction between urban nature and violent crime.

## 2. Materials and Methods 

Using the PRISMA (Preferred Reporting Items for Systematic Reviews and Meta-Analyses) checklist [33], the authors drafted a plan for the literature review. Those articles written in English, published since 2000, and covering research in urban areas (not rural or suburban) within the United States were eligible for inclusion. The independent variable had to include at least one type of green space. At least one of the dependent variables had to be violent crime. No age, sex, socio-economic, health, or gender limitations were placed on study participants. The focus of the search was on original, primary peer-reviewed literature, although doctoral dissertations and master’s theses, white papers, conference proceedings, and articles from organization websites were also eligible. Literature reviews were excluded because they were not primary research. No meta-analysis was undertaken because a range of research types (experimental, quasi-experimental, interventional, non-interventional, qualitative, quantitative, case study, cross-sectional, and longitudinal studies) was eligible for inclusion. Conflicting opinions about the relevance of a particular paper were resolved by a third, independent reviewer. The independent reviewer was blind to who had made the evaluation and the motivations for making the decisions, thus enhancing the validity of the evaluations.

### 2.1. Search Strategy and Database Selection

Databases were selected based on relevance and journal coverage. Subject area specific databases were identified, and a broad interdisciplinary database (Scopus) was also searched. Initial searches took place in December 2017 with updates to the searches run in July 2019. Using the EBSCOhost research platform, a joint search was performed on PsycINFO, Academic Search Premier, and Greenfile. Sociological Abstracts and ProQuest Dissertations and Theses–Global were searched jointly using ProQuest. The results of the searches were de-duplicated using Zotero reference software and then uploaded to Covidence (a systematic review platform), where another round of de-duplication took place. One deviation from the protocol was that title screening for inclusion or exclusion took place ahead of traditional abstract screening because of the large volume of results. Following initial blinded title screening in Covidence, the authors switched to Rayyan, another systematic review platform, for all subsequent screening. Full-text articles emerging from the screening process were evaluated by the authors and eliminated at that stage if they did not meet inclusion criteria.

Search terms were drafted using keywords and terms from papers known to be relevant to the review. Following testing in the chosen databases, the following terms were used in all searches, though the syntax of the search was adapted per database requirements as necessary:

(urban OR cities OR city OR neighborhood OR communit * OR “public housing *”)

AND 

(“green space *” OR green * OR greenspace OR park * OR natur * OR “landscape architecture *” OR “city plan *” OR tree * OR “environment * design” OR ecosystem * OR environment * OR “urban design” OR horticulture OR playground OR garden OR trail OR “urban forestry”)

AND

(crime * OR criminology OR violence OR rape * OR assault OR murder OR aggression OR firearm * OR gun * OR “public safe *”)

### 2.2. Evaluation Process

Two researchers independently screened the titles and abstracts. A third researcher resolved conflicting decisions. Once the final list of papers was established, they were reviewed by the same two researchers to confirm that they were appropriate for inclusion. As previously, when the two paper reviewers disagreed on their evaluation, a third reviewer broke the tie. Once the final list of papers was determined, the three researchers entered summary information into a common spreadsheet, which was later distilled into a literature matrix (see Appendix A).

## 3. Results

### 3.1. PRISMA Summary

**Figure 1:**
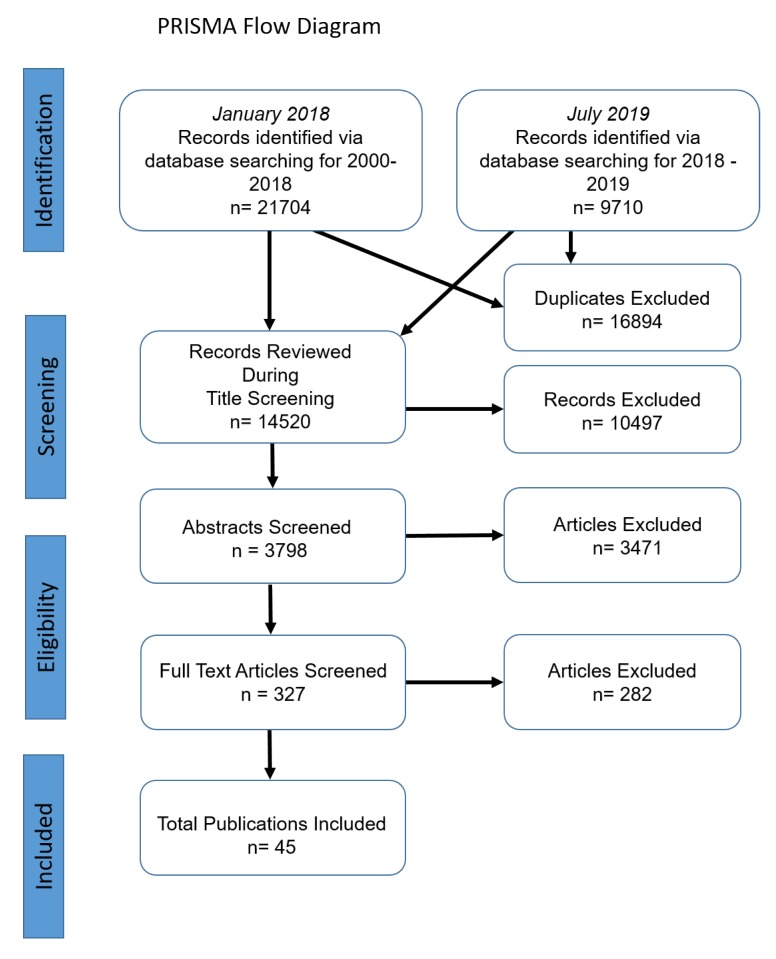
Preferred Reporting Items for Systematic Reviews and Meta-Analyses (PRISMA) flow chart.

In January 2018 and July 2019, 31,414 records were identified via the database search (*n* = 21,704 in 2018 and *n* = 9710 in 2019). Excluding duplicates, 14,520 titles were ultimately screened. After the subsequent title screening, 3798 abstracts remained as potentially relevant publications. After the abstract review, 327 articles were selected for evaluation. Ultimately, 45 papers were selected for inclusion in this study, representing a little over 1% of the original abstracts (see Figure 1).

### 3.2. Patterns in Study Topics and Methods

Green space independent variables fell into five main categories: (1) parks, (2) community gardens and vacant lot remediation, (3) vegetated/tree-lined streets and walkways (including elevated trails), (4) tree canopies and groundcover, and (5) undeveloped or partially developed areas such as ground sewer enhancements, croplands, wetlands, undeveloped nature environments and landscape diversity endeavors (see Table 1).

Criminal behaviors typically addressed by researchers included homicide, assault (including rape), and theft (burglary). Most studies involved both violent personal crime and non-violent property crime (e.g., theft and vandalism). Several studies included disorderly crime, like narcotics use or distribution.

The majority of the 45 selected articles used quantitative methods. Quantitative studies tended to use ArcGIS or other spatial-image analysis tools to assess the presence of parks, vacant lots, or tree canopy. Most studies sought to correlate GIS/image data with jurisdictional crime data. Studies involving tree cover were correlational because almost all employed GIS or image-related data instead of interventions. A few studies predicted causality by employing before and after greening interventions (e.g., greening blighted lots, installing an elevated trail). 

Much of the literature we reviewed and decided to omit was anecdotal, although we were interested in high-quality qualitative studies. Only a handful of studies used qualitative methods. Branas et al., Blair, and Garvin et al. employed a mixed-methods approach by combining quantitative crime analyses with interviews or surveys to assess perceptions of crime and neighborhood disorder [34,35,36]. While most studies included in the scope of this review used jurisdictional crime data, a few research teams used survey measures to assess the risk of aggressive behavior or perceptions of crime and safety.

When considering the methodological challenges shared between these studies, homicide and forcible rape were excluded from the analysis of violent crime in some studies (e.g., Wolfe and Mennis [37]), as these incidents are relatively few. In the case of rape, the data is questionable for other reasons; researchers noted that measuring the frequency of rape is misleading due to the low levels of formal reporting [38].

### 3.3. Study Findings

For studies involving a large range of violent crimes, the most consistent results aligning nature interventions and crime reduction were among studies involving vegetated streets and walkways. As might be expected, the majority of all studies were correlational, and the quasi-experimental studies involving greening interventions were typically limited to community gardens and site greening interventions, likely due to the scale of these projects. Several notable exceptions include the street/walkway improvements research described by Locke et al. [39] and the lot improvements performed by Branas et al. in Philadelphia [11,34,40].

As indicated in Table 1, of the 26 studies addressing all violent crimes, 12 identified a negative relationship between nature and crime (such that crime decreased as nature increased), while four ran contrary to our expectation. Ten studies were deemed inconclusive by their respective authors, as the results did not reach statistical significance or involved a number of confounds. With regard to violent crime (not involving homicide or rape), four of the six relevant studies demonstrated nature’s contribution to crime reduction. The single study specifically focused on homicide [41] found reductions in homicide in parks, although the results regarding the impact of remediated sites were inconclusive. 

Studies that focused specifically on gun violence support the hypothesis that green space reduces this violence [11,34,36,40,42,43,44,45,46]. Of the nine studies (two reported in a single publication by Branas et al. [11]), six had the expected outcomes. Three of the 45 studies were from the same team of researchers (Branas, Kondo, South) who investigated the potential link between green space and crime through the cleaning and greening of vacant lots, primarily in Philadelphia, PA.

#### 3.3.1. Parks

Ten studies addressed the relationship between parks and crime, though all of these studies were correlational and did not study a specific intervention. Three studies found that the presence of parks was associated with reductions in crime, two were inconclusive, and three demonstrated trends in the opposite direction (Abu-Lughod [47], Kim and Hipp [48], and McCord and Houser [49]). Abu-Lughod found that violent crime increased as the number of city-owned parks increased while Kim and Hipp and McCord and Houser suggest that areas near parks experienced higher levels of crime and disorder. These findings may be explained by Jane Jacobs’ “eyes on the street” theory [50] and C. Ray Jeffrey’s Crime Prevention through Environmental Design (CPTED) principles [51], such that an open public place where strangers may be less easily identified by members of the community may create opportunities for crime. McCord and Houser support this explanation, specifically addressing the guardianship theory in their study.

Overall, regarding parks, there are insufficient studies to reflect on the impact on violent crime (not homicide or rape), homicide only, and gun violence. None of the studies in these categories involved interventions, possibly due to the construction cost of a large park intervention and the delays in the development of landscape growth once a park was in place.

#### 3.3.2. Community Gardens/Greening

A larger number of studies (*n* = 12) addressed community gardens and greening of lots. All of these studies suggested that greening interventions or the presence of community gardens were related to a reduction in crime. Included in this group is a series of pre-post studies by Branas et al. in which researchers “cleaned and greened” a series of lots over several years in Philadelphia, PA, resulting in decreased incidence of gun violence [11,34,40]. Heinze et al. [52], Kondo et al. [53], and Sadler et al. [54] also reported similar results from vacant lot greening interventions. The overall positive effects of blighted lot remediation (compared to the mixed results from parks) may also be attributed to CPTED and defensible space theories, such that the removal of abandoned buildings and overgrown brush reduces the amount of shelter and improves visual guardianship of an area.

Interventions (*n* = 7) were most common in this category, likely due to the lower fiscal and physical challenges associated with creating community gardens and greening—they are easier to add to the urban fabric. Branas et al. suggest that their interventions in Philadelphia were inexpensive, scalable, and readily executed in low-income residential areas [11].

#### 3.3.3. Vegetated Streets and Walkways

Although we found a limited number of studies on the impact of vegetated streets and walkways (*n* = 6), all of them support the hypothesis that this type of green space influences crime. Four of the six studies involving vegetated streets found decreases in crime, while crime remained unchanged in the Auchincloss [55] and Locke [39] et al. studies. Auchincloss et al. suggest greenways require associated comprehensive social interventions in order to be effective. Locke et al. raised concerns surrounding the spillover effects, such as a reduction in crime around greened streets may simply spread to perimeter areas. Indeed, Branas et al. echoed this concern in their blighted lot greening studies [11]. Null findings may also be the result of poor operationalization of green space or selection bias in the greening process such that community partners may have not been random in their choice of streets on which to plant trees [39].

Included in the group are four quasi-experimental studies (Auchincloss et al. [55], Harris et al. [56], Harris [57], and Crewe [58]). Quasi-experimental studies were particularly common in this category, as most were pre–post studies examining the influence of a newly established greenway on crime in surrounding areas, including Philadelphia’s 58th St Greenway (Auchincloss et al.), Chicago’s Bloomingdale Trail (Harris et al.), and Boston’s Southwest Corridor (Crewe).

#### 3.3.4. Trees and Ground Cover

The largest number of studies fell into the category of trees and ground cover (*n* = 14), perhaps due to ease of analysis from readily available GIS data. Aerial GIS information can provide detailed information on large-scale urban vegetation and may allow for examination over time due to the natural growth of vegetation. As such, most studies were correlational—none of the studies involved large-scale greening interventions, but they often involved substantially larger datasets than studies in the previous categories. Many papers reported results from geographic and crime data involving entire cities, including Austin, Baltimore, Chicago, Milwaukee, New Haven, Philadelphia, and Portland. Donahue used GIS tools to investigate tree cover in over 200 cities (and 59 individual communities within one city) [59].

The majority of the papers described in this category reflect decreases in crime (*n* = 9), with four others reporting inconclusive results. Of the inconclusive, two revealed nuances in the relationship between urban green space and crime. In their investigation of population density and crime, Lim found a significant moderating effect of vegetation on crime rates, such as high vegetation buffering the influence of high density on violent crime, providing support for cognitive restoration theories [60]. Li also observed a moderated relationship, such as view-blocking vegetation being associated with more violent crime but less property crime [61]. Just as Auchincloss et al. suggested, greenway interventions must be accompanied by appropriate policy changes [55]. Donahue also provides evidence for the importance of implementation plans accompanying urban tree cover interventions [59].

#### 3.3.5. Undeveloped Green Areas (and Other) 

Studies by Kondo et al. [45] and Sparks [62] did not demonstrate significant or conclusive relationships. They were placed under this heading due to the uniqueness of the independent variables that they measured. The Kondo study focused on green stormwater infrastructure and the Sparks study focused on land use diversity such as wetlands, forested land, agricultural land, and barren land. The inclusion of these studies highlight the methodological challenges and nuances associated with green space studies, as the operationalization of greenspace can take many different forms.

## 4. Discussion

### 4.1. State of the Research

Research on the impact of green space crime is limited. Among the prominent findings of this hybrid review was that potentially confounding variables are rarely addressed in detail. This challenge may be related to the lack of a mutually agreed upon grounded theory linking independent, covariate, and depending variables. Relatedly, a prominent conclusion when reviewing the study findings is that, with the exception of a few studies (e.g., Branas et al. [34]), there is insufficient work involving the qualitative analysis that might support the development of a unifying grounded theory.

Future studies will have to emphasize the role of confounding variables or package their independent variables using the concept of bundles, an approach borrowed from medicine, in which a variety of variables are clustered to achieve greater efficiency [63]. In this approach, multiple environmental attributes are thought to produce an outcome, although the impact of a single contributor might not be clear.

In this context, we recommend a variety of future studies, including future research directions recommended within the papers included in this literature review:

- Meta-analyses that aggregate data from multiple research projects, empirical and quasi-empirical.
- Studies that focus on the mechanisms that may be impacting behavioral responses [37,53,56,64,65,66].
- Intervention studies at multiple scales (from small green oases to extensive parks and greenways), particularly those that involve longitudinal pre/post field experiments [11,48,49,55,67,68].
- More studies that exploit the benefits of the development of grounded theory and the gathering of qualitative data, particularly survey and interviews [69].
- More studies that focus specifically on the most violent of crimes—gun violence [11].

### 4.2. Mediators Contributing to the Relationship Between Greenspace and Violent Crime

**Figure 2:**
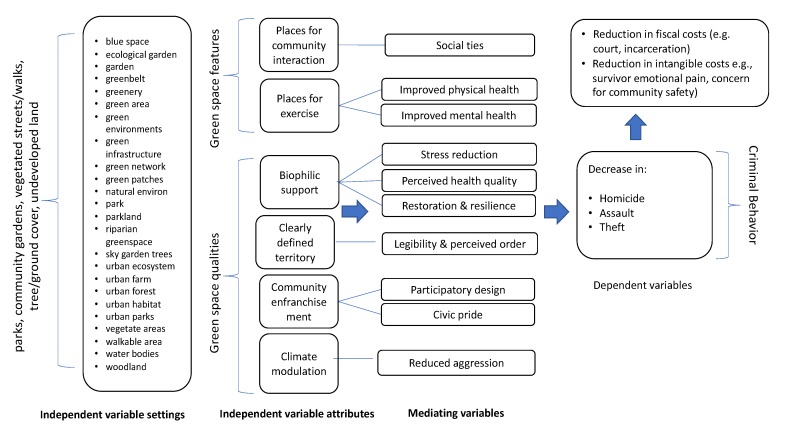
Green space and crime variable relationships.

As mentioned in the previous section, one of our recommendations involves a more thorough understanding of the mediating variables in the interest of determining causality. We assume that the positive impact of green spaces on crime reduction is attributable to the co-presence of multiple factors that can be divided into physical features (places for community interaction and places for exercise) and qualities (biophilic support, territorial definition, community enfranchisement, and climate moderation; see Figure 2). There are undoubtedly additional factors, but these clusters were most prevalent in the literature and are discussed in the following section.

#### 4.2.1. Places for Community Interaction

*Social ties.* Outdoor gathering spaces provide the opportunity for interaction among neighborhood members, which increases familiarity and mutual investment in well-being. In keeping with the theory of collective efficacy, greened lots may promote social cohesion and, as a result, the interest in acting for the common good, thereby normalizing healthy behavior in these spaces [70].

Kuo et al. [71] explored the issue of how individuals’ natural environments relate to their tendency to establish neighborhood social ties. Their study focused on Chicago public housing units that had direct access to common spaces with varying levels of vegetation. The researchers found a correlation between resident perception of “greenness” and strong neighborhood social ties. In addition, Kuo et al. found that “greenness” of common spaces was associated with perceived neighborhood safety.

With specific regard to children and adolescents, readily visible outdoor recreational spaces provide the opportunity for youth activities and potentially deter gang violence. Researchers have found that the presence of recreational amenities geared toward youth reduces the frequency of criminal activities in this age group [72]. Similarly, playgrounds can provide the opportunity for children to learn social and developmental skills [73], which may help them function more effectively in groups, and ancillary parent interaction has the potential for community adhesion through shared childcare activities.

#### 4.2.2. Places for Exercise

*Improved physical health*. Parks provide the opportunity for exercise, which may enhance mental acuity [74] and reduce obesity [75]. Improved cognitive skills and health may enhance judgment. Lack of safety, however, may inhibit physical activity and is associated with fear of violence, presence of concerning behaviors, lack of maintenance and good lighting, and the presence of traffic [76]. Han et al. note that gun crimes are associated with long-term negative impacts on health due to reduced use of parks, in addition to the short-term impacts on public safety [77].

*Improved mental health*. Urban life may be a source of high stress levels [78], and stress and depression are related [79]. The associated mental illness may result in violent behavior. However, exercise is known to produce serotonin and as a result, act as a stress reducer [80]. Parks and other green spaces provide the opportunity for physical activity, including walking, jogging, and playing sports, and, therefore, may contribute to improved mental health.

#### 4.2.3. Biophilic-Related Support

*Stress reduction*. Nature, in and of itself, may have a calming impact on human psychological and emotional state and cognitive functioning. Higher cortisol levels have been reported in urban areas with a higher percentage of green space [81].

*Perceived quality of life*. The presence of parks may increase the perceived quality of life [82], particularly as quality of life concerns the provision of perceived choice and control. Reduced lack of choice and control may mitigate the need to strike out against society and engage in violent activities.

*Restoration and resilience*. Kaplan [83] and others have demonstrated the impact of experience in nature on mental restoration. The resulting ability to make healthier and more productive decisions may be improved by interactions with nature. These interactions may also result in greater resilience [84].

#### 4.2.4. Clearly Defined Territory

*Ownership legibility*. Clearly defined territories lead to less ambiguity of ownership. Replacement of underdeveloped sites with green spaces is a way to establish territorial markers. The simple act of replacing a run-down, unsupervised lot with a community-developed green space may force sites that previously afforded unhealthy activities to relocated or diminish. The lack of “intrinsic ownership” blurs accountability for maintenance and guardianship [85].

*Perceived order*. Wilson and Kellings’ (1982) theory of “broken windows” suggests that minor cases of disorder create a foundation for more serious crime [86]. This disorder might express itself in the form of visual chaos (garbage, graffiti, abandoned cars) [87]. Several studies associate perceived disorder to physical decline, depression, psychological distress, and perceived powerlessness (e.g., Geis and Ross [88]). The implication is that residents see disorder as an indication of a more problematic neighborhood condition with the potential of compromising health [87]. (The socially controversial underside of this approach is linked to philosophies of crime control that recommend the aggressive arrest of individuals for minor infractions.) At the same time, there is considerable concern around gentrification. Upgrades should be supported and developed by the community and in keeping with local cultural aesthetics [11].

#### 4.2.5. Community Cohesion

*Community enfranchisement*. Design researchers have known for many years that the participation of users (community members and clients) in the development of guidelines for the physical environment results in greater acceptance of the space and higher levels of maintenance. User participation has been noted for its particular effectiveness in urban settings [89] and provides opportunities for community members to coalesce around common goals. Community cohesion is a primary predictor of reductions in violent crime [90].

*Civic pride*. Another factor that may contribute to crime reduction is the impact of presence of parks on civic pride [91]; communities that are provided green space amenities may interpret this intervention as an act of respect and collaboration from civic governments. With regard to the duration of exposure, the impact is likely to occur even after short daily interactions with nature [92]. Quality parks may help motivate community members to protect and care for these spaces and reduce the need to erode the physical quality of these facilities as an expression of frustration.

#### 4.2.6. Climate Modulation

*Reduced aggression*. Among the many ecological benefits of trees and other green features is the reduction of the heat island effect [93]. At the same time, researchers have provided evidence that aggression increases in higher ambient temperatures up to certain levels (i.e., 90 degrees Fahrenheit) [94]. The heat-reducing impact of green space, therefore, may result in reduced crime.

### 4.3. Limitations

The initial literature search yielded a substantial number of results (14,520 titles reviewed) due to the nature of the language used in the search. Keywords like “green” and “environment” are used broadly outside of the focus topic. To ensure an inclusive set of studies, the researchers relied on manual weeding (title reviews), potentially introducing researcher bias. This will likely continue to be a significant challenge for other researchers seeking to find articles on the topic of violence and green space within such a large collection of literature.

While the search was comprehensive, it was limited to articles written in English and research that took place in the United States. However, corroborating results have been found in other countries, such as Australia and the United Kingdom [92,95]. The researchers were also challenged by the differing definitions of green space and lack of common methods for calibrating green content. We were also unable to incorporate unreported or in-progress studies.

Discerning the impact of confounding variables posed another challenge, particularly in considering the role of maintenance. Beyond the green features of a space, the design and maintenance of a space can also influence its use and perceptions of safety [96]. The presence of green space has the potential to reduce urban crime, but these findings may be substantially moderated by good design and consistent maintenance.

## 5. Conclusions

Based on the 45 quantitative and qualitative papers summarized here, we can deduce that the presence of parks and other green space reduces urban crime. In the process of our review, we extracted multiple mechanisms from the literature that may account for the impact of green space on crime, including social interaction and recreation, community perception, biophilic stress reduction, climate modulation, and spaces expressing territorial definition. Among the recommendations for future research are a meta-analysis of existing data and the development of grounded theory through qualitative data-gathering methods.

There are several strategies for reducing crime in the U.S. [97], and the provision of green space is one of them. Good public spaces support desirable behaviors and inappropriate public spaces provide the opportunity for increases in criminal behavior, which can be economically costly to society [11,98]. Additionally, safe, accessible green spaces enhance physiological and psychological human health and well-being [99,100,101]. By providing evidence that access to nature has a mitigating impact on violence in urban settings, city governments and communities are empowered to support these interventions.

## Figures and Tables

**Table 1:** Literature Review Matrix by Predictors and Outcomes.

	All Violent Crime		Violent Crime (Not Homicide or Rape)	Homicide Only	Gun Violence
**Parks**	Abu-Lughod (‘06) ° ↑ Blair et al. (‘17) ° ~ Brown (‘18) ° ↓	Lee (‘13) ° ~ McCord et al. (‘17) † ↑ Nitkowski (‘17) ° ↓	Boessen et al. (‘18) ° ~ Kim et al. (‘18) ° ↑	Culyba et al. (‘16) ° ↓	DeMotto et al. (‘06) ° ~
**Community gardens/greening**	Blair (‘14) † ♦ ~ Blair et al. (‘17) ° ~ Gorham et al. (‘09) ° ♦ ~ Heinze et al. (‘18) Δ ↓	Kondo et al. (‘16) Δ ↓ Sadler et al. (‘17) Δ ↓ Wilcox et al. (‘13)		Culyba et al. (‘16) ° ~	Branas et al. (‘11) Δ ↓ Branas et al. (‘16) Δ ↓ Branas et al. (‘18) Δ ♦ ↓ Garvin et al. (‘13) Δ ♦ ~
**Vegetated streets and walkways**	Auchincloss (‘19) † ~ Burley (‘18) ° ↓ ~ Harris et al. (‘18) † ↓	Harris (‘18) † ♦ ↓ Locke et al. (‘17) Δ ~	Crewe (‘01) † ♦ ↓		
**Trees and ground cover**	Donahue et al. (‘11) ° ~ Gilstad-Hayden (‘15) ° ↓ Kondo et al. (‘17-A) † ↓ Kuo et al. (‘01) † ↓	Li (‘08) ° ~ Lim (‘05) ° ~ Schusler et al. (‘18) ° ~ Snelgrove et al. (‘04) ° ↓	Deng (‘15) ° ~ Donovan et al. (‘12) ° ↓ Wolfe et al. (‘12) ° ↓		Troy et al. (‘12) ° ↓ Troy et al. (‘16) ° ↓ Kondo et al. (‘17-B) † ↓
**Undeveloped green areas**	Sparks (‘11) ° ~				Kondo et al. (‘15) Δ ~

Study Design: ° Correlational, † Quasi-experimental (pre-post or control group); Δ Greening intervention; ♦ Included a qualitative component; Findings: ↓ Negative relationship between green space and crime; ↑ Positive relationship between green space and crime; ~ Inconclusive or no significant relationship found; Strictly qualitative studies excluded from this matrix but included in Appendix A.

## Appendix A

**Table A1:** Literature Review Summary Table.

Paper	Study Location; Time	Sample Size; Units	Predictor: Type of Green	Outcome: Type of Crime
Abu-Lughod (2006) [47]	Highest populated cities in US; 2000	n/a	P	H, A, T
Auchincloss et al. (2019) [55]	Philadelphia, PA; 2009–2014	n/a	V	H, A, T
Blair (2014) [35]	Cincinnati, OH; 1997–2011	5 gardens	G	H, A, T
Blair et al. (2017) [64]	Cincinnati, OH; 2013–2014	12 parks and playgrounds; 10 gardens	P, G	H, A, T
Boessen & Hipp (2018) [67]	Nine US cities; n.d.	109,808 blocks	P	H, A, T
Branas et al. (2011) [40]	Philadelphia, PA; 1999–2008	4436 city lots	G	A, T *
Branas et al. (2016) [11]	Philadelphia, PA; 1999–2008	676 buildings	G	A *
Branas et al. (2018) [34]	Philadelphia, PA; 2011–2013	541 vacant lots	G	A, T *
Brown (2018) [102]	Philadelphia, PA and Detroit, MI; 2011–2015	384 and 297 crimes	P	H, A, T
Burley (2018) [65]	Portland, OR; 2011–2015	93 neighborhoods	V	H, A, T
Crewe (2001) [58]	Boston, MA; 1996-1998	2 neighborhoods	V	A, T
Culyba et al. (2016) [41]	Philadelphia, PA; 2008–2014	143 crimes	P, G	H
DeMotto & Davies (2006) [42]	Kansas City, KS; 2002	40 parks	P	H, A *
Deng (2015) [68]	Milwaukee, WI; 2005–2010	1 city	T	A, T
Donahue (2011) [59]	New York, NY; 2001–2008	59 NYC communities (and 200+ other cities)	T	H, A, T
Donovan & Prestemon (2012) [103]	Portland, OR; 2005–2007	2813 households	T	A, T
Garvin et al. (2013) [36]	Philadelphia, PA; 2011	21 lots	G	A, T *
Gilstad-Hayden et al. (2015) [104]	New Haven, CT; 2008–2012	106 census tracts	T	H, A, T
Gorham et al. (2009) [69]	Houston, TX; 2005	11 community gardens	G	T
Harris, Larson, & Ogletree (2018-B) [56]	Chicago, IL; 2011-2015	138 (Study 1) and 62 (Study 2) census tracts	V	H, A, T
Harris (2018) [57]	Chicago, IL; 2011–2015	(see above)	V	H, A, T
Heinze et al. (2018) [52]	Flint, MI; 2009–2013	216 treated lots	G	H, A, T
Kim & Hipp (2018) [48]	Southern CA; 2010	218 cities	P	A, T
Kondo et al. (2015) [45]	Philadelphia, PA; 2000–2012	238 census tracts	U	H, A, T *
Kondo et al. (2016) [53]	Youngstown, OH; 2010–2014	5126 crimes	G	H, A, T
Kondo et al. (2017-A) [105]	Cincinnati, OH; 2005–2014	307 blocks	T	H, A, T
Kondo et al. (2017-B) [46]	Philadelphia, PA; 2008–2011	309 (Study 1) 135 (Study 2) individual victims	T	A *
Kuo & Sullivan (2001) [12]	Chicago, IL; n.d.	98 apartment buildings	T	H, A, T
Lee (2013) [106]	Chicago, IL; 2010	150 parks	P	H, A, T
Li (2008) [61]	Oakland, CA; 2006–2007	234 neighborhoods	T	H, A, T
Lim (2005) [60]	Dallas, TX; n.d.	1683 blocks	T	H, A, T
Locke et al. (2017) [39]	New Haven, CT; 1996–2007	1193 blocks	V	H, A, T
Luke (2013) [107]	Cleveland, OH; 2012	105 gardeners; 92 non-gardeners; 3 community gardens	G	Safety; sense of community
McCord & Houser (2017) [49]	Philadelphia, PA and Louisville, KY; 2005–2010	307 parks	P	H, A, T
Nitkowski (2017) [108]	Milwaukee, WI; 2013–2015	210 census tracts	P	H, A, T
Sadler et al. (2017) [54]	Flint, MI; 2005–2014	1800 lots	G	H, A, T
Schusler et al. (2018) [66]	Chicago, IL; 2009–2013	801 census tracts	T	H, A, T
Seymour et al. (2010) [109]	Los Angeles, CA; 2007	39 individuals	G	Utilitarian relationships with green alleys
Snelgrove et al. (2004) [110]	Austin, TX; 1995	1170 crimes	T	H, A, T
Sparks (2011) [62]	San Antonio, TX; 2003–2006	235 census tracts	U	H, A
Stodolska et al. (2011) [111]	Chicago, IL; 2007	26 crimes	P	Benefits, concerns of parks
Troy et al. (2012) [43]	Baltimore, MD; 2007–2010	1208 census tracts	T	H, T *
Troy et al. (2016) [44]	Baltimore, MD; 2007	999 households	T	H, A, T *
Wilcox et al. (2003) [112]	Seattle, WA; 1989–1990	100 census tracts	G	Impact of parks and playgrounds on crime perceptions
Wolfe & Mennis (2012) [37]	Philadelphia, PA; 2005	363 census tracts	T	A, T

TYPE OF GREEN: ^P^ Parks (larger than community or neighborhood), ^G^ Community greening (alleys, urban gardens, small green space, greening vacant lots), ^V^ Vegetated streets and walkways (elevated trails and street tree planting), ^T^ Trees and ground cover (grass, tree upgrades), ^U^ Less developed green areas (stormwater upgrades, croplands, wetlands, natural spaces, diverse landscaping); TYPE OF CRIME: ^H^ Homicide—General, ^A^ Assault, Sexual Assault, ^T^ Theft, Robbery, Burglary, * Specifies gun crimes.
